# Editorial: Frontiers on innate immunity and intercellular communication

**DOI:** 10.3389/fimmu.2022.1102776

**Published:** 2022-12-19

**Authors:** Yu Xiao, Ying Zhang, Hang Yin, Toshiyuki Shimizu

**Affiliations:** ^1^ Department of Laboratory Medicine, Zhujiang Hospital, Southern Medical University Guangzhou, Guangdong, China; ^2^ School of Pharmaceutical Sciences, Tsinghua University, Beijing, China; ^3^ Graduate School of Pharmaceutical Sciences, The University of Tokyo, Tokyo, Japan

**Keywords:** innate immunity, intercellular communication, extracellular vesicle, TLR, nod like receptor

## Introduction

As one of the important components of the primary defense system, the innate immune system can be rapidly deployed in organisms against microbial infections upon activation. Both pathogen-associated molecular patterns (PAMPs) and damage-associated molecular patterns (DAMPs) activate innate immune pattern recognition receptors to trigger downstream inflammatory responses and other immune responses. Therefore, elaborating more detailed mechanisms for the precise regulation of innate immunity has important implications for the treatment of human diseases.

Various soluble factors and particles in body fluids mediate immunological, long-distance intercellular communication. In the past decades, the investigation of extracellular vesicles (EVs), a group of nano-size particles with lipid bilayer membranes that carry particular cellular contents of parent cells, is a paradigm shift for traditional intercellular communication mechanisms and ushers in a new research interest for cell-to-cell communication mechanisms. EVs may raise the alarm for innate immune responses or silence them *via* the transfer of cargo biomolecules between cells to affect intracellular communication (1).

This Research Topic encompasses a collection of reviews, opinions, perspectives, as well as primary research articles investigating Innate Immunity and Intercellular Communication.

## Exploration of the unknown in innate immunity

Although innate immune signaling pathways have been well investigated, there are still have many aspects in this area that need more in-depth study. The scientific researchers involved in the current Research Topic introduce us to some interesting research directions, pointing towards future research possibilities.

Pathogen constituents, including microbial lipids and nucleic acids (NAs), are able to activate Toll-like receptor (TLR) signaling pathways. To prevent unnecessary autoimmune responses, NA-sensing TLRs are localized in the endosomal compartment. With the degradation of NAs in endosomal compartment, TLR subfamilies are less activated by self-derived NAs. Surprisingly, NA degradation products were found to be effective TLRs ligands. Miyake et al. summarize recent findings of how dysregulation of NA metabolism affects TLR responses and their relationship with diseases.

Unlike the TLR family, nucleotide-binding and oligomerization domain (NOD)-like receptors (NLRs) have different subcellular localization and complex caspase reaction signals. However, limited structural evidence restrained the understanding of NLR activation mechanism. Ohto‘s review introduces the mechanisms of activity regulation and signal transduction as revealed by structural biology studies conducted over the past decade. These detailed structural biology studies are expected to aid further development of therapeutic agents.

Ym1 is a widely confirmed M2 polarization marker, but there is still a lack of research on its function. Kang et al.‘s review highlights the relationship between Ym1 and diseases such as allergic lung inflammation, parasitic infection, autoimmune diseases and nervous system diseases.

Protein tyrosine phosphatases (PTPs) remove phosphates from tyrosine residues in some proteins. Luo et al. found an SH2-domain-containing PTP named LvPTPN6 in *Litopenaeus vannamei* and reveal the mechanism whereby LvPTPN6 enhances the antiviral immunity of shrimp *via* the JAK/STAT signaling pathway.

Extracellular traps (ETs) of neutrophils are widely reported in both animals and plants, where they play a vital role in killing invading microbes. Liao et al.‘s review summarize the mechanism through which bacteria, fungal pathogens and parasites use nucleases to evade the ETs of the host.

Both innate and adaptive immune processes involve hemichannels (HC)/gap junctions (GJs) and immunoglobulin (Ig)-like domain-containing proteins (IGLDCPs). Meng et al. summarize the current understanding of HC-released immune signaling factors that influence IGLDCPs in regulating innate/adaptive immunity.

HMGB1 can function as a DAMP outside the cell, activating the immune system and promoting inflammation. Gao et al. show that HMGB1 mobilization was completely inhibited by selective H1-receptor antagonists, suggesting that histamine induces HMGB1 release from vascular endothelial cells through H1 receptor stimulation. Anti-HMGB1 antibodies may become a new therapeutic option.

## Development of disease therapy strategies

Cannabidivarin (CBDV) is a non-psychoactive phytocannabinoid found in plants of the *Cannabis* species. Wang et al. demonstrate that CBDV has anti-neuroinflammatory effects through direct binding to MD2, possibly acting as an antagonist of TLR4. CBDV further mediates analgesic effects by inhibiting morphine-induced glial activation. CBDV could be a potential agent for improving morphine-mediated analgesia.

ACT001 was proven to be an anti-tumor drug. In Tianshu Zhang et al.‘s work, ACT001 effectively reduced the peripheral injury and activation of microglia and astrocyte *via* inhibiting the formation of TLR4/MD2/MyD88 complex. Their work provides strong evidence for ACT001’s potential in the treatment of neuropathic pain.

## Investigating roles of EVs in innate immunity

EVs are known as a novel carrier of MAMPS and DAMPs. Therefore, studying isolation methods and mechanisms for EVs is helpful for the investigation of the function of EVs in innate immunity.

Although gut bacteria cannot easily cross the complex gut-blood barrier into circulation, a large number of studies have shown that both gram-positive and -negative EVs can be detected in circulation. Wei et al. presents a new bioanalytical technology for the purification of bacterial extracellular vesicles (BEVs), which contain various bioactive compounds and thus can be further used as therapeutic approaches for modulating innate immune responses of host cells. Epsilon-poly-L-lysin (ϵ-PL) is useful for collecting BEVs, and BEVs purified by ϵ-PL (ϵ-PL BEVs) are comparable to those concentrated by ultracentrifugation.

In aging individuals, chronic inflammation is widely observed, but the biological functions of EVs are still beyond investigation. Xiao et al. found that senescent fibroblasts in the aging microenviroments release larger amounts of miR-30b-5p containing EVs. These EVs can specifically activate the classical NF-kB signaling pathway *via* releasing miR-30b-5p into recipient cells.

## Conclusion

The Research Topic of ‘Innate Immunity and Intercellular Communication’ has gathered numerous worthy investigations and contributions on the subject of innate immune signaling pathways and intercellular communication, offering us research tactics and insights into new research direction on new players in signaling pathways. The articles in this collection highlight important interrelated topics and will hopefully serve as a catalyst for further studies such as elucidation of mechanisms involved in signaling pathways in innate immune responses. Ultimately, we trust that these efforts may eventually lead to the development of new anti-inflammatory drugs and bioengineering techniques for theranostics.

## Author contributions

YX and TS completed the draft manuscript. And all authors listed have made a substantial, direct, and intellectual contribution to the work and approved it for publication.
